# Unveiling the Diagnostic Significance of Calprotectin in Serous Ovarian Carcinoma

**DOI:** 10.3390/life15071080

**Published:** 2025-07-08

**Authors:** Alkis Matsas, Eleni Stamoula, Konstantinos Kontzoglou, Makarios Eleftheriades, Sophia Tsokkou, Panagiotis Christopoulos, Theodoros Panoskaltsis, Eleni Polydorou, Ioannis Sotiropoulos, Theodore Troupis, Dimitrios C. Iliopoulos

**Affiliations:** 1 Laboratory of Experimental Surgery and Surgical Research ‘N.S. Christeas’, Medical School, National and Kapodistrian University of Athens, 11527 Athens, Greece; 2 Department of Clinical Pharmacology, School of Medicine, Aristotle University of Thessaloniki, University Campus Aristotle University of Thessaloniki, 54124 Thessaloniki, Greece; 3 Second Department of Obstetrics and Gynecology, Medical School, “Aretaieion” University Hospital, National and Kapodistrian University of Athens, 11527 Athens, Greece; 4 Department of Medicine, Faculty of Health Sciences, Aristotle University of Thessaloniki, 54124 Thessaloniki, Greece; 5 Laboratory of Brain Exosomes and Pathology—ExoBrain Institute of Biosciences and Applications, National Center for Research “Demokritos”, 15341 Athens, Greece; 6 Department of Anatomy, Faculty of Health Sciences, Medical School, National and Kapodistrian University of Athens, 75 Mikras Asias Str, 11627 Athens, Greece

**Keywords:** ovarian cancer serum Calprotectin, CA-125, CA 15-3, inflammatory biomarkers

## Abstract

**Introduction**: Ovarian cancer (OC) is one of the most common gynecologic malignancies and has the highest mortality rate among them. OC has a multifactorial pathogenesis and is characterized by silent onset, progression, and late-stage detection. Therefore, accurate and early detection is of great importance in order to improve survival rates. Emerging evidence reveals that tumor markers are valuable diagnostic and monitoring tools. In this study, we evaluated the aforementioned potential of three markers CA-125, CA 15-3, and serum Calprotectin. CA-125 is a protein that is found elevated in cases of ovarian, breast, and lung cancer. Cancer Antigen 15-3 (CA 15-3) is a protein detected in high levels in women with breast cancer and ovarian cancer and it is significantly elevated in patients with metastasis and recurrence of OC. Calprotectin is a protein released from activated neutrophils, related to inflammatory conditions and can be a potential immune-mediated marker in OC. **Purpose:** The purpose of this study was to explore the significance of serum calprotectin, CA-125, and CA 15-3 in women diagnosed with serous OC. **Methodology**: Thirty-eight (38) women with diagnosed OC were included in this research as the study group and twenty-seven (27) healthy women with no history or current diagnosis of OC were included in the control group. Women in both groups shared similar past histories to avoid any other parameters interfering with the study. Our study group was further subdivided into early stage and advanced stage patients. Blood samples were collected from all women of both groups and were examined using ELISA kits to evaluate the levels of the above markers. **Results**: When comparing patients versus control patients, those with OC exhibited higher levels of Calprotectin compared to healthy individuals. Additionally, Calprotectin showed a statistically significant elevation between the control group and advanced patients. CA-125 remains the current standard of care biomarker exhibiting 90% sensitivity, whereas sensitivities in Calprotectin and CA 15-3 were 60% and 50%, respectively. **Conclusions**: Serum CA-125 remains the single most valuable biomarker for ovarian cancer, having the highest statistical significance, correlation with disease stage, detecting both early or advanced patients, and sensitivity of 90%. It appears to be a promising inflammatory biomarker in the early diagnosis of ovarian cancer, showing an elevation in patients, while CA 15-3 provides moderate complementary information and exhibits inferior sensitivity when compared to both CA-125 and Calprotectin. The latter appears to be a promising marker and further studies could show if its addition to established protocols could improve early detection, disease progression, or risk stratification. Calprotectin enhances the detection range for ovarian cancer when used alongside CA-125, while this combined approach detected a greater proportion of patients than CA-125 alone, indicating improved diagnostic potential.

## 1. Introduction

Ovarian cancer (OC) is the second most common gynecological malignancy in the United States and Western Europe [1] and exhibits the highest rate of mortality [2]. Despite significant advances in gynecologic cancer treatment, OC remains a major contributor to mortality due to its insidious onset, silent progression, and late-stage diagnosis. Approximately 70% of OC cases are diagnosed at an advanced stage, resulting in a 5-year survival of only 30% [3]. OC is responsible for 2.5% of cancers in women. Whilst it is the 11th most common cancer among women, OC is the 5th leading cause of cancer-related death among the female population [4].

Regarding the prevalence of OC and the low survival rate, it is essential to delve into the molecular pathways of ovarian cancer etiopathogenesis. While numerous hypotheses have been advanced to elucidate the etiopathogenesis of OC, four have emerged as particularly prominent and they highlight the intricate interaction between ovulatory, inflammatory, and hormonal variables. The prevailing theory is the incessant ovulation theory, which links OC to repeated ovulatory cycles. Each ovulation activates a proinflammatory cascade and triggers the release of cytokines/chemokines and matrix-remodeling enzymes [5,6]. The core of the second theory (hormonal imbalance theory) that applies to postmenopausal women, is that unopposed estrogen due to estrogen-only replacement therapy contributes to a higher OC risk. The tubal hypothesis, as suggested by Salvador et al., states that the fimbriae of the fallopian tubes is the emerging point of OC development, especially the high-grade serous one, based on the theory of increased cancer risk with tubal exposure to oxidative stress and inflammatory agents due to retrograde menstruation and supported by the presence of serous tubal intraepithelial carcinomas. Lastly, the inflammation hypothesis attributes ovarian cancer to chronic inflammatory processes, where upregulated chemical mediators promote tumor initiation, malignant conversion, and progression [7,8].

Broadly, there are three types of OC based on the different ovarian cells that are involved. More specifically, epithelial cells (type I OC) are the cells detected in the ovarian surface and this category is further divided into many subtypes. Within this group, there are different subtypes, including high-grade serous, low-grade serous, clear cell, endometrioid, and mucinous cancers, while serous ovarian cancer is the most common subtype of epithelial ovarian cancer. Overall, epithelial ovarian cancer is the most common type of ovarian cancer (more than 95% of all OC cases) and nearly 70% of women with this type are diagnosed in advanced stages [9]. The second type consists of germ cell tumors which stem from the primordial germ cells that ultimately develop into ova. Subtypes of this category are yolk sac tumors, immature teratoma, and dysgerminoma. Finally, sex-cord stromal cells comprise the type III ovarian carcinoma, including malignant granulosa and Sertoli–Leydig cells. This type of ovarian cancer derives from the tissue cells that release female hormones [10,11].

Globally, OC is deemed the most lethal gynecological cancer due to its silent progression and late-stage diagnosis. Early and accurate diagnosis is crucial to improve survival outcomes. Tumor markers have emerged as valuable tools in the early diagnosis and management of OC offering non-invasive options for screening and monitoring [12]. Nowadays, tumor markers, also known as oncomarkers, play a significant role in cancer research. Tumor markers are molecules released from malignant cells or cells in their vicinity and can be detected in blood and body fluid. Regarding their molecular origin, they can be antigens, cytoplasmic proteins, hormones, enzymes, oncogenes, and their derivatives [13,14]. Ideally, a tumor marker should possess high specificity and sensitivity to a specific type of cancer, prognostic benefits, clinical validation, and accurate positive and negative predictive values. To date, there is no tumor marker that meets all the aforementioned criteria.

Currently, CA-125 is the most established gynecological oncotumor marker whereas biomarker CA 15-3, although primarily utilized as a breast cancer tumor marker, has been thoroughly investigated in ovarian malignancies as well. CA-125 is a mucin 16-derived protein that is commonly elevated in cases of ovarian, breast, and lung cancer [15,16]. Although approximately 80% of women with advanced epithelial ovarian cancer show elevated CA-125 levels, its sensitivity in early-stage disease is suboptimal. Reported sensitivities are approximately 50% for stage I and about 90% for stage II and higher [17].

One of the major limitations of CA-125 is its high false-positive rate. Elevated serum CA-125 levels can be observed not only in malignancies other than epithelial ovarian cancer but also in about 1% of healthy individuals and approximately 5% of patients with benign conditions such as endometriosis, benign ovarian cysts, and inflammatory pelvic disease [18].

Due to these limitations, the FDA recommends the use of CA-125 primarily for treatment response assessment and monitoring of residual disease, rather than as a sole diagnostic marker [16].

CA 15-3 is a mucin-1-derived protein that is most commonly found elevated in breast cancer patients. It is primarily utilized for monitoring treatment response and assessing disease progression. Serum CA 15-3 levels can also be elevated in other malignancies, including liver, lung, and ovarian carcinomas [19,20]. In the context of ovarian cancer, CA 15-3, when used in combination with CA-125, may enhance sensitivity for early diagnosis. However, CA 15-3 levels are typically elevated in advanced stages and metastatic disease where they show a strong correlation with disease progression and poor clinical outcomes [21,22].

Calprotectin, also known as human leucocyte protein L1 [23,24], MRP 8/14, S100A8/S100A9 complex [25,26], and calgranulin [27], is a zinc- and calcium-binding protein heterodimer. Calprotectin is released due to exocytosis of granules from activated neutrophils and represents a marker of inflammation [28,29,30,31,32]. Intracellularly, Calprotectin exhibits numerous functions including activation of NADPH oxidase, induction of apoptotic pathways, and free-radical scavenging [33,34]. It has antimicrobial and chemotactic activities, as well as proinflammatory cytokine functions [26,35,36].

Emerging research unravels a correlation between Calprotectin and tumor development, but the exact role has not yet been determined. Calprotectin seems to inhibit enzymes involved in cell proliferation and induces intracellular apoptotic pathways [14,26,37]. Additionally, Calprotectin inhibits metalloproteinases, a group of endopeptidases that play a central role in angiogenesis and metastatic tumor growth [38]. Previous studies have revealed that Calprotectin is overexpressed in numerous types of cancer [39,40,41,42]. Elevated Calprotectin levels have also been observed in patients with advanced-stage ovarian cancer, suggesting its involvement in tumor-associated inflammation and immune dysregulation [43].

Since Calprotectin is considered an inflammatory marker, and on the other hand, inflammatory environment through which oxidative stress induces DNA damage is associated with tumor initiation in fallopian tube epithelium, the hypothesis in this study was that there is a link between serum Calprotectin and ovarian cancer development and that it could serve as a novel biomarker in ovarian cancer.

This study aimed to evaluate Calprotectin levels in OC and compare its predictive validity with other well-established diagnostic markers.

## 2. Materials and Methods

### 2.1. Study Population

After the inclusion/exclusion criteria were applied, a total of 65 women were included. The study population (n = 38) comprised of women with early primary serous ovarian carcinoma (stage I) (n = 18) and women with advanced primary serous ovarian carcinoma (n = 20), (stages II, III, IV). The remaining women were subsequently registered in the control group (no cancer present).

### 2.2. Participants

Women under the age of 40 and over the age of 65 were not included in this study. Additional exclusion criteria included a final histopathological diagnosis indicating a benign or borderline tumor, a tumor of non-ovarian origin, or a histological subtype other than serous. Individuals who declined to participate were also excluded. Moreover, patients receiving immunosuppressive therapy, those on long-term nonsteroidal anti-inflammatory drugs (NSAIDs), hormone replacement therapy, or oral contraceptives were not eligible. Patients with a known history of inflammatory bowel disease, hepatic, renal, autoimmune, thyroid, or hematological disorders were also excluded. Finally, patients with a prior diagnosis of any other malignancy, a recent infection or inflammation, or current pregnancy were not included in this study.

### 2.3. Procedure

After surgical excision, tumors were examined by a pathologist for the final histopathological analysis, diagnosis, grading and staging (I–IV) according to International Federation of Gynecology and Obstetrics (FIGO) standards. They were then classified into three groups based on their histopathological staging. The first group (group A) consisted of 18 women with early primary serous ovarian cancer (stage I) whereas the second group (group B) consisted of 20 women with advanced serous (stages II, III, IV). Group C (Control group) included 27 healthy women with no pathology known and no history of any form of cancer nor a family history of breast or gynecological cancer. Both healthy women and patients participating in the trial underwent a blood testing as part of the pre-operative evaluation. Through the blood examination CA-125, CA 15-3 and Serum Calprotectin levels were assessed.

### 2.4. Sample Collection and Determination CA-125, CA 15-3 and Calprotectin

Blood samples were collected via serum separator vials and centrifuged as per the manufacturers’ instructions following their coagulation. More specifically, they were centrifuged for 20 min at 1000× *g* at 2–8 °C, transferred to Eppendorf tubes and then stored at −80 °C (<3 months). Serum CA-125 levels were determined using ALINITY I CA-125 II CAL assays (catalog number: 08P4901) on the Alinity I instrument (Abbott, AbbottPark, Chicago, IL, USA). Serum CA15-3 levels were measured using Alinity I CA 15-3 CAL assays (catalog number: 08P5101) on the Alinity I instrument (Abbott, AbbottPark Chicago, IL, USA). Serum CALP (Calprotectin) levels were measured by ELISA kits (catalog number: E-EL-H2357, Elab science Biotechnology, Houston, TX, USA). All measurements were performed according to the manufacturers’ recommendations.

### 2.5. Ethics and Approval

The study protocol was approved by the institutional bioethics board of the National and Kapodistrian University of Athens (institutional review board number 506). All participants provided written informed consent for study participation. This study was conducted according to the Declaration of Helsinki and samples were collected from all the patients who were initially detected via transvaginal ultrasound (TVUS) and subsequently via iodine-based contrast enhanced magnetic resonance imaging (MRI) with a clinically suspicious ovarian cystic mass or solid adnexal mass. Additionally, patients’ body weight and height were obtained with calibrated devices and body mass index (BMI) was calculated. Clinical information was collected from hospital records and by additional patient interviews.

### 2.6. Statistical Analysis

The independent samples Kruskal–Wallis test was used to assess differences between the three groups, as sample size restrictions did not allow for the conduction of parametric tests. Non-parametric post hoc multiple comparison tests followed to verify statistically significant pairwise comparisons between groups adjusting for type I error inflation. The Spearman’s Rho correlation coefficient was used to examine correlations between biomarkers. To account for multiple pairwise comparisons, Dunn’s test was used. ROC curves were applied to examine the diagnostic accuracy of the three markers, and their comparison was based on the Delong test. The analysis was carried out using Jamovi v2.3.26 and the statistical significance was set at 0.05 in all cases.

## 3. Results

**Figure 1:**
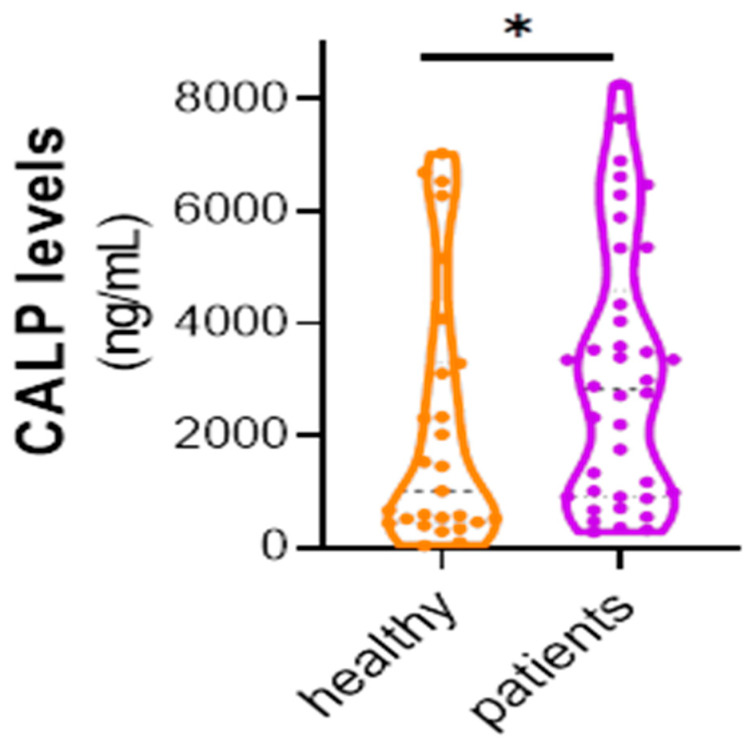
CALP levels (ng/mL) distribution between healthy individuals (n = 27) and patients (n = 38).

We measured serum levels of Calprotectin (CALP) using a commercially available ELISA kit. Our sample consisted of 27 healthy individuals marked as controls and 38 patients. We found that patients exhibit higher levels of CALP compared to healthy individuals (Mann–Whitney test: U = 362, *p* = 0.0446, two-tailed) (Figure 1).

Our sample consisted of 20 advanced CA patients, 18 early-stage CA patients, and 27 controls, with a mean age of 54.8 years (SD = 8.2). Statistically significant differences were found between the three groups for all outcomes, specifically CALP, CA-125, and CA 15-3, as well as in correlation to age and BMI (Table 1). Statistically significant lower values were observed in the control group compared to the two CA groups regarding CA-125 (*p* < 0.001) and CA 15.3 (*p* = 0.003 for the advanced CA group and *p* = 0.035 for the early CA group). Regarding CALP, statistically significant lower values were observed in the control group versus the advanced CA patients (*p* = 0.016), while the difference between the two CA groups was not statistically significant (*p* = 0.120).

**Age**

Lower age values were found in the control and early-stage groups compared to the advanced group with *p* < 0.001 and 0.031, respectively, as described by the non-parametric multiple comparisons tests (MCTs) and the weighted probability estimate (adj. Sig, Table 2).

**BMI**

Lower BMI values were found in the control group compared to the advanced patientsgroup (*p* = 0.03) as shown by the non-parametric multiple comparisons tests (MCTs) and the weighted probability estimate (adj. Sig, Table 3).

**CALP**

**Figure 2:**
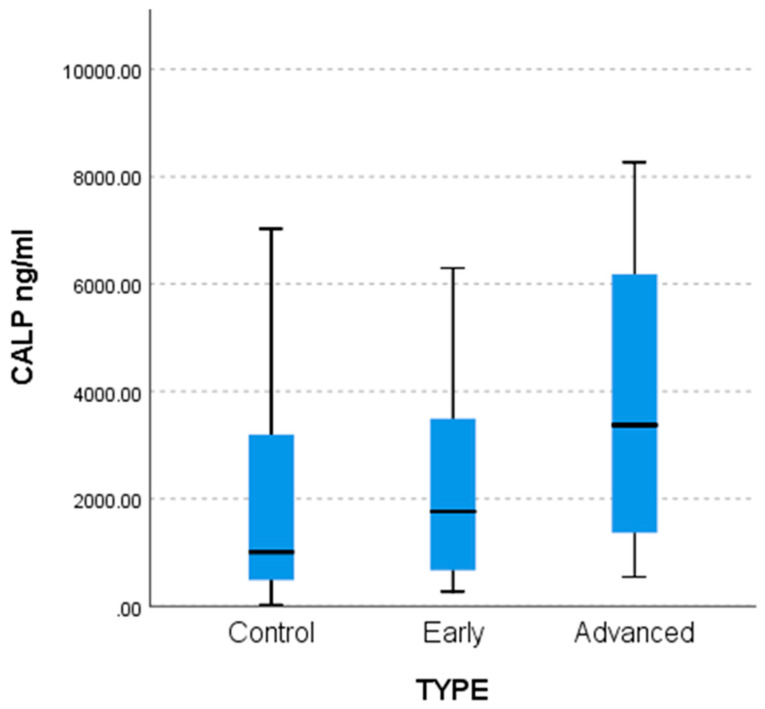
Difference between control (n = 27), early (n = 18), and advanced (n = 20) patients for CALP values.

Statistically significantly lower Calprotectin (CALP) values were found in the control group compared to the advanced group (*p* = 0.016) as shown by the non-parametric multiple comparisons tests (MCTs) and the weighted probability estimate (adj. Sig, Table 4). The differences are captured by the comparative bar chart in Figure 2 below.

**CA-125**

**Figure 3:**
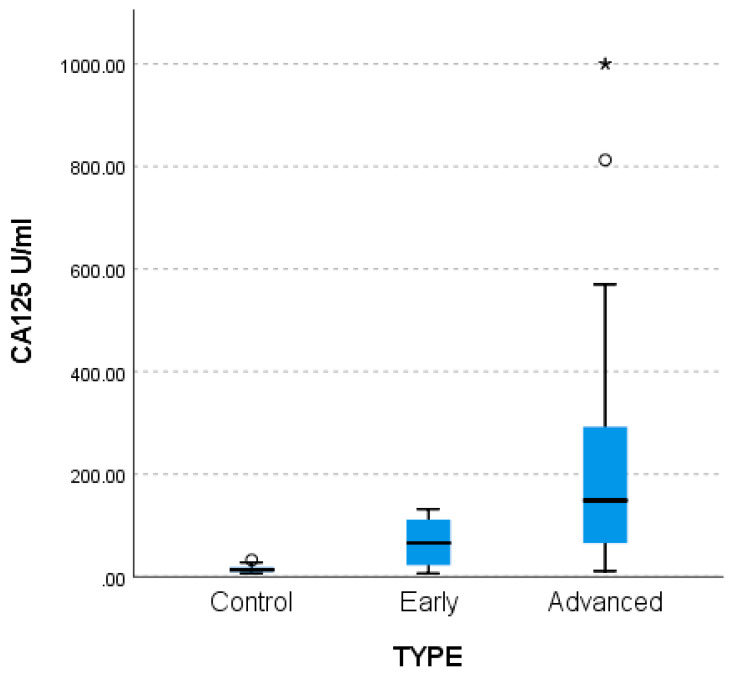
Difference between control (n = 27), early (n = 18), and advanced (n = 20) patients for CA-125.

Statistically significantly lower CA-125 values were found in the control group compared to the two patient groups (*p* < 0.001) as shown by the non-parametric multiple comparisons tests (MCTs) and the weighted probability estimate (adj. Sig, Table 5). The differences are described in the comparative bar chart in Figure 3 below.

**CA 15-3**

**Figure 4:**
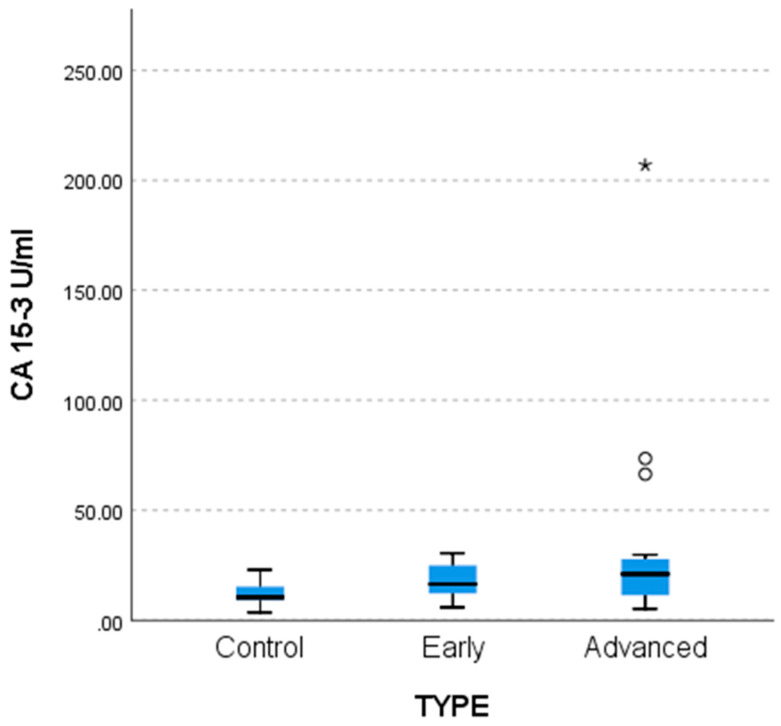
Difference between control (n = 27), early (n = 18), and advanced (n = 20) patients regarding CA 15-3.

Lower CA 15-3 values were found in the control group compared to the two patient groups (*p* = 0.003 for advanced and 0.035 for early stage) as shown by non-parametric multiple comparisons tests (MCTs) and by the weighted probability estimate (adj. Sig, Table 6). The differences are reflected in the comparative bar chart in Figure 4 below.

### 3.1. ROC Analysis

Statistically significant correlations using the Spearman Rho correlation coefficient were found between CA-125, CA15-3 and CALP (Table 7). The correlation between CA-125 and CA 15-3 and CALP were highly significant but moderately strong, while the correlation between CA 15-3 and CALP was weak with a *p*-value equal to 0.038 (Table 8). All three correlations were positive.

**Figure 5:**
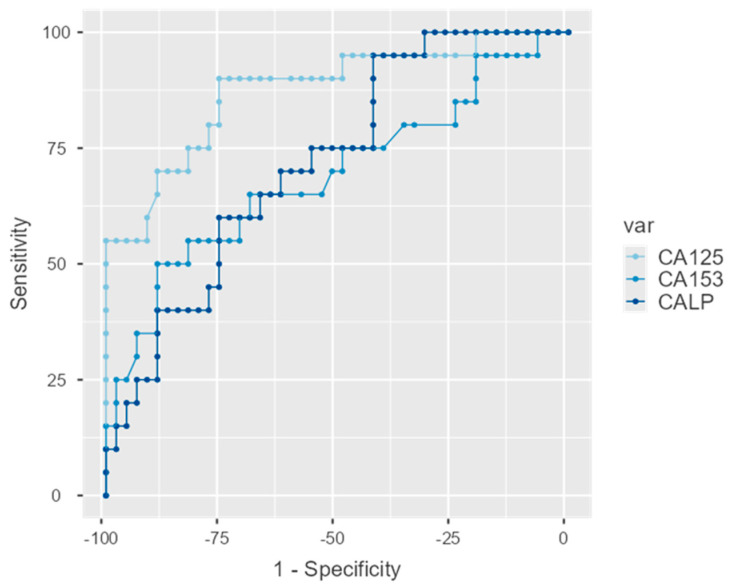
Comparative ROC curve estimation.

ROC curves were estimated for the three biomarkers and were statistically significant in all cases with detailed estimations appearing in Table 8. The AUC for CA-125 was equal to 0.875 (95%C.I.: 0.777–0.973; *p* < 0.001) for CA 15-3 0.691 (95%C.I.: 0.540–0.842; *p* = 0.014) and for CALP 0.721 (95%C.I.: 0.594–0.849; *p* = 0.005). These are graphically displayed in Figure 5.

The DeLong test of difference between AUCs indicated differences between CA-125 and the two other indices (*p* = 0.013 in both cases) which do not differ between them.

### 3.2. Sensitivity and Specificity

Regarding CA-125, sensitivity was equal to 90% and specificity equal to 76% for a cutoff value of 35 up to 38. Lower scores were observed for CA 15-3 with sensitivity equal to 50% and specificity equal to 89% for a cutoff value of 23 and for CALP with sensitivity equal to 60% and specificity equal to 76% for a cut off value of 3321.

## 4. Discussion

In this study, we tried to elucidate the clinical use and importance of Calprotectin as a possible diagnostic biomarker in the early diagnosis of ovarian cancer. Calprotectin has attracted substantial academic interest regarding its role in various cancers, lymphomas, and inflammatory conditions [44,45,46,47]. The latest evidence also implements its diagnostic potential to certain conditions or diseases like inflammatory bowel diseases or even systemic infection and sepsis [48,49].

Our findings are in accordance with the existing body of evidence, reinforcing Calprotectin as a potential biomarker for OC. Previous studies in the field of gynecologic oncology have recently shown that plasma Calprotectin levels have been found to be significantly increased in ovarian cancer patients compared to women with benign ovarian tumors, proposing its diagnostic relevance. Current literature includes only a limited number of clinical studies regarding the use of serum Calprotectin as a potential early biomarker in epithelial ovarian cancer, while only two studies include its use specifically for serous ovarian cancer diagnosis, highlighting the importance of this study and what it has to offer to the existing knowledge.

We used strict inclusion criteria for our study. Participants were eligible only if they met all of the established criteria, namely age, health status, absence of chronic diseases or any signs of inflammation, and zero use of drugs, including any type of anti-inflammatory drug, immunosuppressants, hormone replacement therapy, or oral contraceptives.

A study included 199 women with either ovarian carcinomas, borderline ovarian tumors (BOT), or benign ovarian tumors serving as the control group. The plasma Calprotectin concentration and the serum CA-125 concentrations were measured among subjects and were found to be elevated, especially in the carcinoma patients group, suggesting that these markers rise in parallel with tumor burden or related inflammatory processes. According to the researchers, the CA-125 marker was found to be superior to Calprotectin while also showing higher sensitivity for diagnosing ovarian malignancy [50]. These results are also in accordance with our study where serum levels of Calprotectin (CALP) appeared to be elevated in patients compared to the control group, reaffirming its established diagnostic role.

In this study, we advanced our analysis by separating patients into two groups, namely stage 1 patients described as early patients and stage 2, 3, or 4 described as advanced patients. We found CA-125 to be a superior marker showing statistically significant elevation between both early or advanced patients and the control group. Calprotectin (CALP) showed a statistically significant elevation between the control group and advanced patients but not with early patients. No statistically significant elevation was found between early and advanced patients. It is important to note that our analysis regarding the CALP biomarker showed a *p* = 0.04, significant before adjustment, but not significant after the correction, denoting the need for a targeted or larger sample size study.

A different study signified the importance of early detection of ovarian cancer in its prognosis and management. Their findings align with those reported by Ødegaard et al. and this study. According to the researchers, increased serum Calprotectin protein levels were observed in a small cohort of women with ovarian neoplasia compared to those with benign ovarian cysts [27].

Another study explored the role of Calprotectin as a marker in endometrial cancer regarding outcome, therapy, and disease recurrence. The researchers used healthy premenopausal and postmenopausal women as control groups and found a strong positive correlation between Calprotectin concentration and endometrial cancer [51]. Furthermore, in a study, researchers measured Calprotectin concentrations during gynecological cancer and underlined the correlation between inflammation and cancer. They included both ovarian and uterine cancer, with the former showing the biggest elevation in Calprotectin levels [52]. Many studies have also invigorated this hypothesis. Neutrophils can enhance cancer cell growth, metastasis, and tumor angiogenesis by secreting cytokines and chemokines, playing an important role in immune response, contributing significantly to tumor development [53]. As the tumor microenvironment includes inflammatory cells, with a high concentration of pro-inflammatory cytokines, their existence has been correlated to tumor initiation, progression, and metastasis in many malignancies, including ovarian cancer, indicating the need for further studies [54,55]. In addition to inflammation, incessant ovulation has also been linked to an increased risk of high-grade serous carcinoma (HGSC), as ovulation has been interpreted as an increase in inflammation through elevated oxidative stress and high cytokines, chemokines, and bradykinins secretion [56].

Certain studies also indicate the importance of CA 15-3 as a biomarker in gynecological malignancies diagnosis. A study found a link between CA 15-3 levels and residual tumor after surgery, disease progression, and response to the treatment [22]. In this study, we also measured the levels of CA 15-3 in all groups. We found statistically significant elevation between healthy individuals and both early or advanced patients, while no difference was observed between the two patient groups. Based on the three different biomarkers that we measured, we tried to elucidate which one showcases the highest sensitivity and specificity. Our ROC analysis estimations for CA-125, CA 15-3, and CALP as described by the area under the curve (AUC) showed that CA-125 is the strongest biomarker followed by CALP and lastly by CA 15-3. Our finding matches a study comparing the AUCs of CALP and CA-125 where the latter also appeared to be superior. The AUC of 0.875 for CA-125 was comparable to values reported by Odegaard et al. (AUC ≈ 0.85) [50]. Despite the recognized importance of CA-125 as a biomarker, the number of undetected cases and their poor outcome necessitates the use of new biomarkers in combination to the existing ones, as even a few additional early detected cases could be very beneficial in the management and prognosis of the disease.

This study has certain limitations. A bigger sample size could show stronger evidence towards the utilization of these biomarkers or any difference between them. It is important to note though that we implemented strict inclusion criteria in this study, regarding our control group, minimizing confounding variables while also achieving a more homogenous study population. Our target population regarding patients was also very restricted with very few individuals meeting the eligibility requirements. Also, the limited literature on the use of these biomarkers in the diagnosis and management of ovarian cancer impact external validity but also highlight the importance of new knowledge.

## 5. Conclusions

This study confirms that serum CA-125 remains the single most valuable biomarker for ovarian cancer, having the highest statistical significance, correlation with disease stage, detecting both early or advanced patients, and ROC AUC. It appears to be a promising immune-mediated marker in ovarian cancer, showing an elevation in patients, while CA 15-3 provides moderate complementary information. Calprotectin (CALP) appears to be a promising inflammatory marker in OC and further studies could show if its addition to established protocols could improve early detection or risk stratification. This study demonstrates that Calprotectin, as a novel biomarker, widens the detection window for ovarian cancer when combined with CA-125. This combination approach identified a higher percentage of patients compared to using CA-125 alone, suggesting improved diagnostic potential.

## Figures and Tables

**Table 1:** Differences between the three groups in patients’ characteristics and outcomes.

			Mean	SD	Median	Range	N	KW	*p*
AGE	TYPE	Control	50.44	7.73	49.00	25.00	27	18.539	<0.001
		Early	54.50	7.94	57.50	25.00	18		
		Advanced	60.80	4.93	62.50	22.00	20		
BMI	TYPE	Control	24.28	3.83	23.23	15.36	27	7.076	0.029
		Early	26.16	4.07	26.37	15.72	18		
		Advanced	27.48	5.10	27.36	21.27	20		
CA-125 U/mL	TYPE	Control	14.88	6.44	13.30	25.60	27	35.550	<0.001
		Early	68.92	45.75	65.80	125.17	18		
		Advanced	269.80	316.15	148.60	989.10	20		
CALP ng/mL	TYPE	Control	2156.22	2299.80	1005.76	6997.92	27	8.259	0.016
		Early	2155.62	1735.04	1763.64	6025.60	18		
		Advanced	3858.95	2521.40	3371.72	7724.56	20		
CA 15-3 U/mL	TYPE	Control	11.62	5.11	10.50	19.30	27	12.350	0.002
		Early	17.41	7.41	16.45	24.60	18		
		Advanced	32.46	44.69	21.05	201.70	20		

**Table 2:** Multiple comparisons tests between the three groups regarding the age of healthy individuals or patients.

Sample 1–Sample 2	Test Statistic	Std. Error	Std. Test Statistic	Sig.	Adj. Sig.
Control–Early	−8.167	5.744	−1.422	0.155	0.465
Control–Advanced	−23.886	5.569	−4.289	0.000	0.000
Early–Advanced	−15.719	6.133	−2.563	0.010	0.031

**Table 3:** Multiple comparisons tests between the three groups regarding the BMI value of healthy individuals or patients.

Sample 1–Sample 2	Test Statistic	Std. Error	Std. Test Statistic	Sig.	Adj. Sig.
Control–Early	−9.574	5.753	−1.664	0.096	0.288
Control–Advanced	−14.374	5.578	−2.577	0.010	0.030
Early–Advanced	−4.800	6.143	−0.781	0.435	1.000

**Table 4:** Multiple comparisons tests between groups regarding CALP.

Sample 1–Sample 2	Test Statistic	Std. Error	Std. Test Statistic	Sig.	Adj. Sig.
Control–Early	−2.926	5.753	−0.509	0.611	1.000
Control–Advanced	−15.543	5.578	−2.786	0.005	0.016
Early–Advanced	−12.617	6.143	−2.054	0.040	0.120

**Table 5:** Multiple comparisons tests between groups regarding CA-125.

Sample 1–Sample 2	Test Statistic	Std. Error	Std. Test Statistic	Sig.	Adj. Sig.
Control–Early	−20.370	5.753	−3.541	0.000	0.001
Control–Advanced	−32.523	5.578	−5.831	0.000	0.000
Early–Advanced	−12.153	6.143	−1.978	0.048	0.144

**Table 6:** Multiple comparisons tests between groups regarding CA 15-3.

Sample 1–Sample 2	Test Statistic	Std. Error	Std. Test Statistic	Sig.	Adj. Sig.
Control–Early	−14.519	5.752	−2.524	0.012	0.035
Control–Advanced	−18.230	5.577	−3.269	0.001	0.003
Early–Advanced	−3.711	6.142	−0.0604	0.546	1.000

**Table 7:** Spearman Rho correlation coefficient between CA-125, CA15-3, and CALP.

		CA 15-3 U/mL	CALP ng/mL
**Spearman’s Rho**	**CA-125 U/mL**	**0.480**	**0.392**
	**CA 15-3 U/mL**		**0.258**

**Table 8:** ROC analysis estimations for CA-125, CA 15-3, and CALP.

Test Result Variable(s)	Area	Std. Error	Asymptotic Sig.	Asymptotic 95%C.I.	
				Lower Bound	Upper Bound
CA -125 U/mL	0.875	0.050	0.000	0.777	0.973
CA 15-3 U/mL	0.691	0.077	0.014	0.540	0.842
CALP ng/mL	0.721	0.065	0.005	0.594	0.849

## Data Availability

The data presented in this study are available with in the article.
